# Giant inguinal hernia, a case series

**DOI:** 10.1016/j.ijscr.2025.112040

**Published:** 2025-10-10

**Authors:** Anupam K. Gupta

**Affiliations:** Good Samaritan Hospital, 5 Good Samaritan Way, Mt. Vernon, IL, 62864, USA

**Keywords:** Inguinal hernia, Giant Inguinal hernia, Compartment syndrome, Huge inguinal hernia

## Abstract

**Introduction and importance:**

Inguinal hernia surgery is common worldwide. A giant inguinal hernia occurs when the hernia extends below the midpoint of the thigh, making reduction into the abdominal cavity challenging and posing significant operative and postoperative risks.

**Case presentation:**

We report a case series of five male patients, aged 51–75 years, with giant inguinal hernias treated between January 2024 and January 2025. Four patients presented electively and one emergently with strangulation. All hernias extended below the midpoint of the thigh. Open surgical repair was performed in all cases, with reduction via an abdominal incision above the inguinal crease. The hernia contents included small and large bowel loops with omentum. In one case, the inferior epigastric artery was ligated to facilitate reduction.

**Clinical discussion:**

We report a case series of five male patients, aged 51–75 years, with giant inguinal hernias treated between January 2024 and January 2025. Four patients presented electively and one emergently with strangulation. All hernias extended below the midpoint of the thigh. Open surgical repair was performed in all cases, with reduction via an abdominal incision above the inguinal crease. The hernia contents included small and large bowel loops with omentum. In one case, the inferior epigastric artery was ligated to facilitate reduction.

**Conclusion:**

Giant inguinal hernias require individualized surgical planning. Careful reduction, sac excision, and reinforcement with mesh can achieve good outcomes. Ligation of the inferior epigastric artery may aid reduction in specific cases. A better understanding of socioclinical factors leading to delayed presentation may improve earlier detection and intervention.

## Highlights

• Giant inguinal hernias are rare, usually due to long-term neglect and delayed presentation.
• Socioeconomic, cultural, and occupational factors contribute to delayed diagnosis.
• Surgical management requires individualized planning, sometimes including arterial ligation.
• No patients in this series developed abdominal compartment syndrome.
• Most patients recovered well, with seroma being the only complication.

## 1 Introduction

Inguinal hernia is a common surgical condition characterized by a defect in the groin area leading to extrusion of abdominal contents [1]. Groin hernias may be inguinal, femoral, or obturator in origin [1,2]. Inguinal hernias are classified as direct or indirect based on their relation to the inferior epigastric artery [[1], [2], [3]]. Incarcerated or strangulated hernias may present with pain, nausea, vomiting, and obstipation, requiring urgent surgery [3,4].

When the hernia sac extends below the midpoint of the inner thigh in a standing position, it is defined as a giant inguinal hernia [7]. These hernias usually develop over many years, often due to neglect or barriers to accessing care. Giant inguinal hernias can contain substantial amounts of bowel and omentum, are usually incarcerated, and can present electively or as emergencies [8].

The importance of reporting such cases lies in the rarity, complexity of management, and need for sharing strategies with the surgical community. The estimated prevalence is low, with fewer than 0.5 % of inguinal hernia presentations classified as giant in available literature.

We present a case series of giant inguinal hernias and our management approach, along with a comparison to published literature.

## 2 Case series

This work is reported in line with the PROCESS criteria [9].

Between January 2024 and January 2025, five patients with giant inguinal hernias presented to our surgical department (Table 1). All five patients had hernias descending below the midpoint of the thigh (Fig. 2) for a prolonged duration and had not sought medical care. All were male, aged 51–75 years, with body mass indices ranging from 19.29 to 35.85. Three patients had a long-term smoking history and chronic obstructive pulmonary disease (COPD).

**Table 1:** Patient details.

Case	Age/Sex	ASA score	BMI	Smoking	Comorbidity	Other hernias	Side	Type	Contents	Presentation	Repair	Reduction	Discharge	Complications
1	67/M	4E	19.29	Positive	COPD, CHF	None	Left	Direct	Bowel + omentum	Emergency	Primary	Manipulation	Day 2	Seroma
2	63/M	3	27.73	Positive	COPD, CAD, HTN, HL	Umbilical, epigastric	Right	Indirect	Bowel + omentum	Elective	Primary + Mesh	Manipulation	Same day	Seroma (drainage)
3	74/M	3	35.8	Positive	COPD	None	Right	Direct	Bowel + omentum	Elective	Primary + Mesh	Inferior epigastric artery ligation	Same day	Seroma
4	57/M	3	34.85	Negative	HL, HTN	Hiatal hernia	Right	Direct	Bowel + omentum	Elective	Primary + Mesh	Manipulation	Same day	None
5	51/M	3	35.03	Negative	None	None	Right	Indirect	Bowel + omentum	Elective	Primary + Mesh	Manipulation	Same day	None

COPD chronic obstructive pulmonary disease, CHG congestive heart failure, CAD coronary artery disease, HTN hypertension, HL hyperlipidemia.

**Fig. 1:**
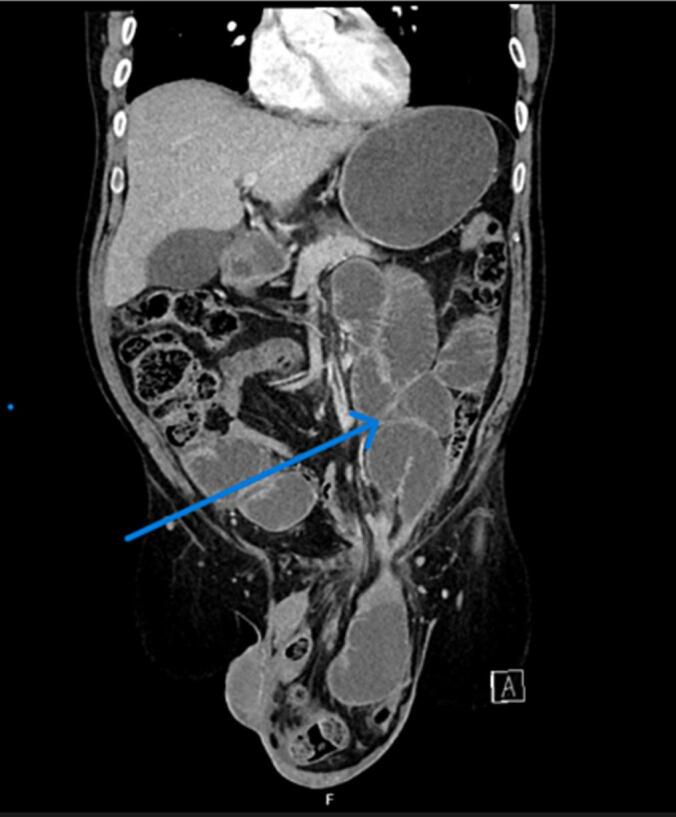
Giant inguinal hernia with intestinal obstruction with dilated proximal bowel loops.

Only one patient presented as an emergency with features of strangulation (Fig. 1). Four of the five patients had right-sided inguinal hernias; three were direct and two were indirect. The hernia sac contents in all cases included bowel loops, omentum, and thickened hernia sac. All hernias were incarcerated and could not be reduced even under anesthesia (Fig. 3).

**Fig. 2:**
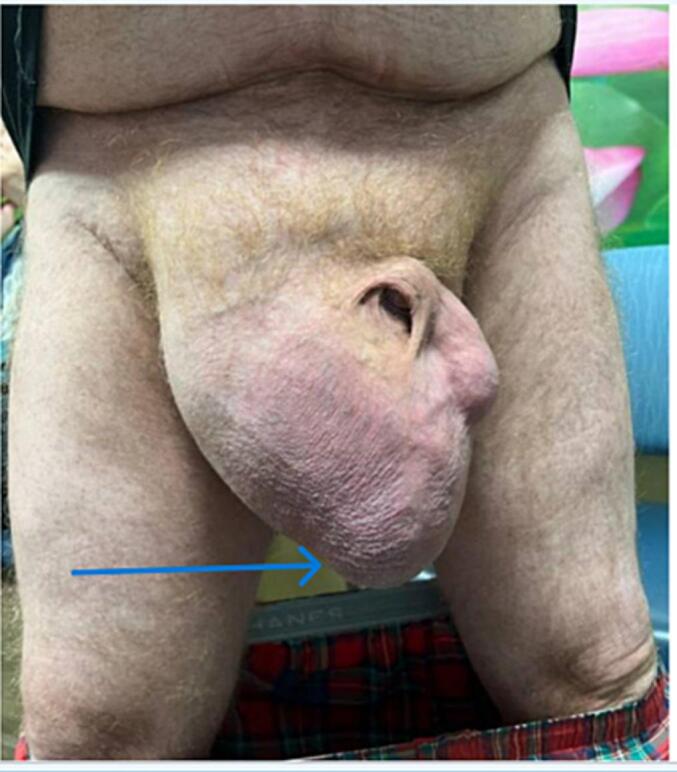
Giant inguinal hernia in standing position.

**Fig. 3:**
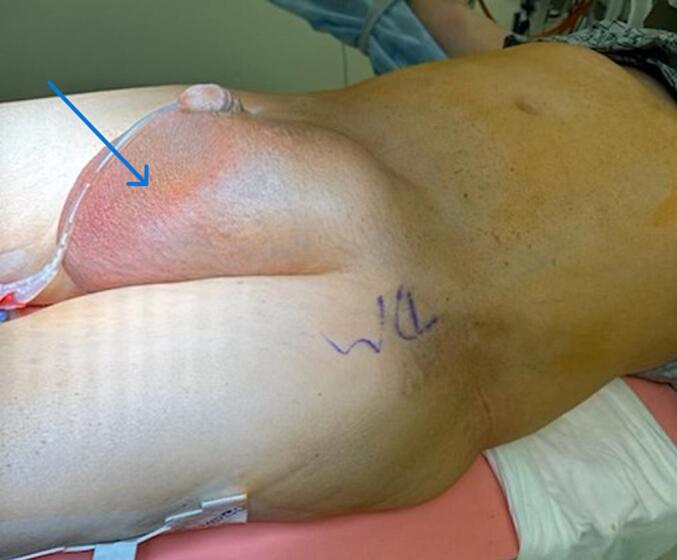
Hernia not able to be reduced under anesthesia.

**Fig. 4:**
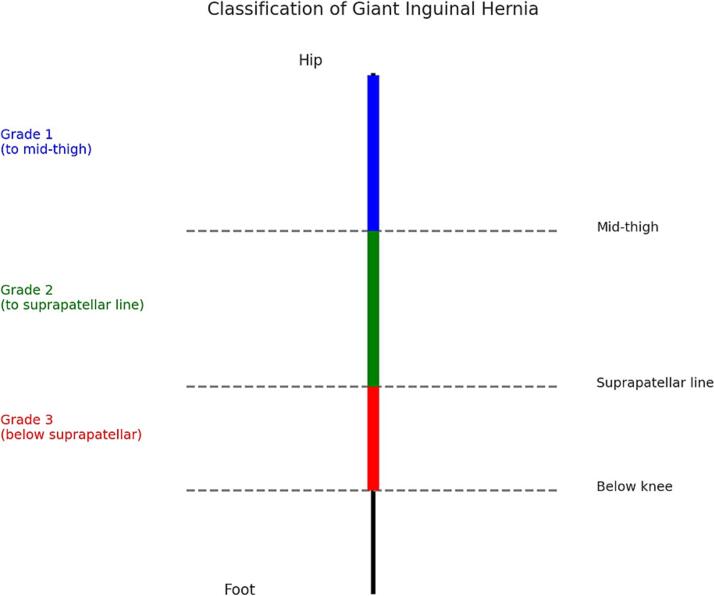
Illustrates the classification of giant inguinal hernias by grade.

All patients underwent an open surgical approach with an incision above the inguinal crease. The hernia contents were reduced back into the abdominal cavity in four of five patients. In one patient with a direct right-sided inguinal hernia, the inferior epigastric artery was ligated to widen the hernia neck. This was achieved by taking down the internal oblique and transverse abdominal muscles, allowing reduction of the hernia contents. All patients had hypertrophied sacs, which were excised to the level of the inguinal ring or the floor of the inguinal canal.

Despite all five being giant inguinal hernias, contents were reduced successfully, and the defect was closed with mesh in four patients. In the emergency case, the bowel loops were congested but viable, and resection was not required after releasing the constriction at the neck of the sac. Due to concern for bacterial translocation and potential future mesh infection, mesh was not used in this patient.

None of the patients developed postoperative abdominal compartment syndrome or ileus, despite significant handling of bowel loops. Four patients were discharged on the same day, while the emergency case was discharged on postoperative day two. Three patients developed seromas: Case 2 required drainage via a scrotal incision, while Cases 1 and 3 were managed conservatively. No recurrence was observed during the follow-up period of 6–12 months. There were no mortalities or severe complications.

## 3 Discussion

Based on the size, giant inguinal hernias are graded as 1, 2, or 3 when examined in the standing position. Grade 1 extends to the mid-thigh, Grade 2 extends between the mid-thigh and suprapatellar line, and Grade 3 extends below the suprapatellar line (Fig. 4). When hernias become very large, it can be surgically challenging to reduce the contents into the abdomen [10]. The surgical planes and anatomy are distorted due to stretching of cord structures, hypertrophy of cremasteric fibers, and hypertrophy of the sac, as well as variations in sac contents [10,11]. There have been no large case series that standardize management [10,11]. Various surgical techniques have been described depending on size, presentation, and contents [12]. Some reports describe bowel, mesentery, or omentum resection to reduce contents into the abdomen. Others describe combined inguinal and midline abdominal incisions to reduce contents and identify anatomy [[11], [12], [13]]. Postoperative compartment syndrome has also been reported, sometimes requiring decompression laparotomy [13,14].

In our case series, we reported only type 1 giant inguinal hernias. We were able to reduce them gradually by manipulation and sac excision. After reducing the contents, we excised the large hypertrophied sac at the level of the inguinal ring or the floor of the inguinal canal. This reduced intra-abdominal volume and may help prevent increased compartment pressures. Intra-abdominal pressures were monitored during and at the end of the procedure to rule out compartment syndrome.

In one case, reduction was aided by taking down the inferior epigastric artery and lateralizing the cord structures. The inferior epigastric artery, easily identified at the root of the sac, was ligated to gain additional space at the hernia neck. The internal oblique and transverse abdominis muscles were mobilized laterally toward the anterior superior iliac spine, which created more space to reduce the contents. This avoided a midline incision and allowed subsequent mesh reinforcement [14,15].

Patients with giant hernias often present late due to socioclinical reasons. Limited access to healthcare, financial difficulties, occupational demands such as manual labor, fear of surgery, and gradual adaptation to symptoms may all delay presentation [8,[10], [11], [12], [13], [14], [15]]. In our series, most patients lived with their hernias for many years before seeking care. Three had a long history of smoking and COPD, which further complicated progression. These factors highlight the importance of understanding patient behavior and addressing barriers to early treatment.

## 4 Limitations

This case series is limited by its small sample size, single-center design, and relatively short follow-up (6–12 months). Long-term recurrence rates cannot be assessed. Detailed occupational and socioeconomic history was incomplete for some patients. Future multicenter studies with longer follow-up are required. We recommend that less experienced surgeons consider stepwise reduction, sac excision, and—in select cases—ligation of the inferior epigastric artery to facilitate safe repair.

## 5 Conclusion

Giant inguinal hernias are rare, usually long-neglected, and surgically challenging. Individualized approaches, including careful manipulation, sac excision, and mesh reinforcement, are effective. Ligation of the inferior epigastric artery can facilitate reduction in selected cases. Recognizing socioclinical barriers is essential for earlier diagnosis and prevention.

## Author contribution

**Anupam Kumar Gupta:** Conceptualization, Methodology, Data curation, Writing – original draft and revisions.

## Consent

Written informed consent was obtained from all patients for publication of this case series and accompanying images. A copy of the written consent is available for review by the Editor-in-Chief of this journal on request.

## Ethical approval

Ethical approval was not required for this retrospective case series as it describes anonymized patients treated as part of standard surgical care without any deviation from standard management. Written informed consent for publication of their clinical details and images was obtained from all included patients.

## Guarantor

**Anupam K Gupta** is the guarantor of this work and accepts full responsibility for the conduct of the study, had access to the data, and controlled the decision to publish.

## Research registration number

NA; This study does not report a “First in Man” procedure or novel intervention requiring registration. It is a retrospective case series of patients managed with standard surgical techniques.

## Funding

This research did not receive any specific grant from funding agencies in the public, commercial, or not-for-profit sectors.

## Funding

No funding was received for this study.

## Conflict of interest statement

The authors declare no conflicts of interest.
